# Pre-Exposure Prophylaxis (PrEP) as an Additional Tool for HIV Prevention Among Men Who Have Sex With Men in Belgium: The Be-PrEP-ared Study Protocol

**DOI:** 10.2196/resprot.6767

**Published:** 2017-01-30

**Authors:** Irith De Baetselier, Thijs Reyniers, Christiana Nöstlinger, Kristien Wouters, Katrien Fransen, Tania Crucitti, Chris Kenyon, Jozefien Buyze, Céline Schurmans, Marie Laga, Bea Vuylsteke, Be-PrEP-Ared Study Group

**Affiliations:** ^1^ Department of Clinical Sciences Institute of Tropical Medicine Antwerp Belgium; ^2^ HIV and Sexual Health Unit Department of Public Health Institute of Tropical Medicine Antwerp Belgium; ^3^ HIV/Sexually Transmitted Infection Clinic Department of Clinical Sciences Institute of Tropical Medicine Antwerp Belgium; ^4^ Department of Medicine University of Cape Town Cape Town South Africa; ^5^ See Authors' Contributions section for collaborators/group members

**Keywords:** pre-exposure prophylaxis, HIV prevention, MSM, Belgium, daily, event-driven, demonstration project, acceptability, adherence

## Abstract

**Background:**

Pre-exposure prophylaxis (PrEP) is a promising and effective tool to prevent HIV. With the approval of Truvada as daily PrEP by the European Commission in August 2016, individual European Member states prepare themselves for PrEP implementation following the examples of France and Norway. However, context-specific data to guide optimal implementation is currently lacking.

**Objective:**

With this demonstration project we evaluate whether daily and event-driven PrEP, provided within a comprehensive prevention package, is a feasible and acceptable additional prevention tool for men who have sex with men (MSM) at high risk of acquiring HIV in Belgium. The study’s primary objective is to document the uptake, acceptability, and adherence to both daily and event-driven PrEP, while several secondary objectives have been formulated including impact of PrEP use on sexual behavior.

**Methods:**

The Be-PrEP-ared study is a phase 3, single-site, open-label prospective cohort study with a large social science component embedded in the trial. A total of 200 participants choose between daily or event-driven PrEP use and may switch, discontinue, or restart their regimen at the 3-monthly visits for a duration of 18 months. Data are collected on several platforms: an electronic case report form, a Web-based tool where participants register their sexual behavior and pill use, a more detailed electronic self-administered questionnaire completed during study visits on a tablet computer, and in-depth interviews among a selected sample of participants. To answer the primary objective, the recruitment rate, (un)safe sex behavior during the last 6 months, percentage of reported intention to use PrEP in the future, retention rates in different regimens, and attitudes towards PrEP use will be analyzed. Adherence will be monitored using self-reported adherence, pill count, tenofovir drug levels in blood samples, and the perceived skills to adhere.

**Results:**

All participants are currently enrolled, and the last study visit is planned to take place around Q3 2018.

**Conclusions:**

As PrEP is not yet available in Belgium for use, this study will provide insights into how to optimally implement PrEP within the current health care provision and will shape national and European guidelines with regard to the place of PrEP in HIV prevention strategies.

**ClinicalTrial:**

EU Clinical Trial 2015-000054-37; https://www.clinicaltrialsregister.eu/ctr-search/trial/2015-000054-37/BE (Archived by WebCite at http://www.webcitation.org/6nacjSdmM).

## Introduction

Pre-exposure prophylaxis (PrEP) using Truvada (emtricitabine/tenofovir disoproxil fumarate) is a promising addition to the field of HIV prevention. Several clinical trials among men who have sex with men (MSM) at high risk of HIV infection have shown that daily PrEP is effective in preventing HIV when taken correctly [1-3]. Within the European context, the UK-based PROUD clinical trial examined daily use, whereas the French Ipergay study tested event-driven PrEP (ie, on demand, before and after anticipated sex). Both trials yielded significant protection effects (86% reduction in incident HIV) [4,5], showing that PrEP is a very promising tool to prevent HIV within this high-risk population.

Daily Truvada use for PrEP was approved by the Food and Drug Administration (FDA) in the United States as early as July 2012 [6]. As of September 2015, the World Health Organization (WHO) recommended that people at substantial risk of HIV infection should be offered PrEP as part of combination prevention approaches [7]. The European HIV prevention landscape is also changing rapidly. In November 2015, daily and event-driven Truvada as PrEP, in combination with safer sex practices, was approved in France [8]. After recommendation by the European Medicines Agency (EMA), the European Commission approved once-daily Truvada as PrEP in combination with safer sex practices in August 2016 [9,10]. Norway joined France by providing PrEP free of charge to at-risk groups in October 2016, and the United Kingdom will make PrEP available in the context of a large clinical study in mid-2017 [11,12]. While European PrEP guidelines are available, they provide little detail and remain general, and large-scale implementation guidelines are lacking [13]. Therefore context-specific experiences with PrEP delivery that can help to shape appropriate recommendations are urgently needed.

The number of MSM in Belgium is estimated to be around 106,000, which is 4.2% of the total male population [14]. As in many western European countries, MSM represent a high-risk population for both HIV and other sexually transmitted infections (STIs) including gonorrhea, syphilis, and chlamydia infection. In 2013, 61% of all registered STIs in men in Belgium were reported among MSM (sentinel surveillance) [15]. Since 2002, there is a trend of increasing numbers of new HIV infections among MSM, who represented 50% of the 1001 newly registered HIV infections in Belgium in 2015 [16]. A venue-based, cross-sectional study was conducted in 2009-2010 among 649 MSM in 2 Flemish cities in Belgium, Antwerp and Ghent, and revealed HIV prevalences as high as 14.5% in cruising venues to 4.9% in more general gay venues to 1.4% at younger MSM venues [17].

PrEP is a potential game-changer for the HIV epidemic among MSM in Western Europe including Belgium, but little is known about how PrEP will be used and how different regimens will influence sexual behavior and lifestyles of MSM.

The overall aim of this study is to provide the necessary data to shape Belgian and European guidelines with regard to the place of PrEP in strengthening HIV prevention. To this end, the following primary and secondary study objectives have been formulated.

The primary study objectives are as follows:

- To document the current preventive needs of MSM at high risk of acquiring HIV, including the uptake, acceptability, and feasibility of using PrEP daily or event-driven
- To evaluate adherence to the 2 different PrEP regimens

The secondary objectives can be found in Textbox 1.

**Secondary objectives of the Be-PrEP-ared study.**

- To study the impact of pre-exposure prophylaxis (PrEP) use on other preventive strategies such as condom use
- To study the impact of PrEP use on sexually transmitted infection (STI) trends
- To study the safety of daily and event-driven use of PrEP
- To document real-life effectiveness of PrEP use on HIV seroconversion and treatment-related resistance
- To evaluate the feasibility of 3-monthly HIV testing using oral fluid self-sampling testing

## Methods

### Study Design

The Be-PrEP-ared project is a single-site, open-label prospective cohort study with a nested qualitative component. It takes place in the HIV/STI clinic of the Institute of Tropical Medicine (ITM), Antwerp, Belgium. A total of 200 MSM at high risk of acquiring HIV were enrolled and are being followed up for 18 months, with 3-monthly follow-up (FU) visits. Truvada is being provided to them as part of a comprehensive HIV prevention package including regular HIV/STI testing and basic adherence and sexual health counseling.

### Participants

Eligibility is assessed using the inclusion and exclusion criteria as shown in Textbox 2.

**Selection criteria of the Be-PrEP-ared study.**

Inclusion criteria:

- Able and willing to provide written informed consent
- Born to male sex (including transgender females)
- Aged 18 years or more
- Had sex with a man in the last 12 months
- HIV negative (confirmed at enrollment)
- Reporting at least 1 criterion for high risk:
  - Condomless anal intercourse in the last 6 months with a casual partner with unknown HIV status or HIV positive status
  - A sexually transmitted infection episode in the last 6 months
  - Having taken post-exposure prophylaxis in the last 6 months
- Able and willing to participate in the project as required by the protocol for 18 months
- Motivated to strengthen prevention efforts, including willingness in starting to use pre-exposure prophylaxis

Exclusion criteria:

- Having symptoms or clinical signs consistent with acute HIV infection
- Being allergic to the active substances or any of the excipients
- Having an estimated creatinine clearance of <60 mL/minute/1.73 m² according to the CKD-Epi formula (Chronic Kidney Disease Epidemiology Collaboration)
- Having an active hepatitis B infection
- Taking HIV post-exposure prophylaxis
- Participating in other clinical studies (phase I-III) or another research project related to HIV and antiretroviral therapy

### Sample Size

Given the provided funding, we included 200 MSM at high risk of acquiring HIV. The sample size was determined to estimate the proportion of participants who are nonadherent. Using the normal approximation for the calculation of the 95% confidence interval and using the worst-case value for proportion of nonadherence of 0.5 with 200 participants, the proportion of nonadherence can be estimated to a precision of 7%.

### Participant Recruitment

The project has been advertised by websites of various community-based gay and sexual health organizations and their respective social network sites and person-to-person promotion of the project. Referral to the project is also done by health care providers at the HIV/STI clinic at ITM. Potential participants were invited to preregister on the project website [18] where details about study participation were also provided. Registered candidates were then invited for a screening visit at the clinic in random order so that they were not invited in the same order as they registered with the exception of the last candidates, who were invited on a “first come first served” basis.

### Investigational Product

Truvada is used for PrEP in this trial. One daily film-coated tablet contains 200 mg of emtricitabine and 245 mg of tenofovir disoproxil fumarate.

**Figure 1:**
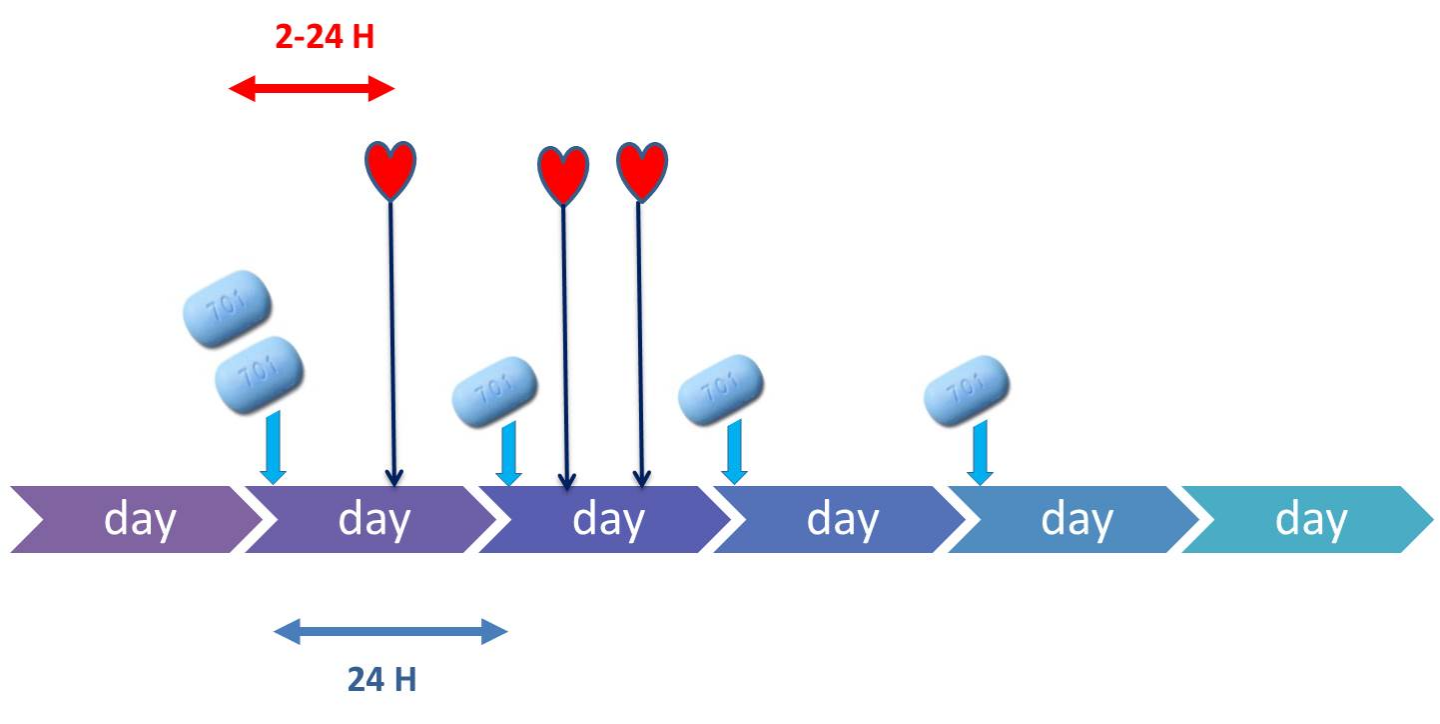
Event-driven scheme (adapted from the Ipergay protocol).

When eligible, participants can self-select between 2 different PrEP dosing regimens:

- A pill every 24 hours (further referred to as daily)
- Starting dose before anticipated sex and 1 pill daily during a sexual active episode (further referred to as event-driven). This is the regimen which has been described by the Ipergay protocol (Figure 1) [4]:

- A dose of 2 pills between 2 and 24 hours before having sex (or 1 pill if the most recent dose was taken between 1 and 6 days ago)
- A tablet of Truvada every 24 hours (starting when the first 2 tablets are taken) during the period of sexual activity including after the last sexual intercourse
- Finally, a last dose of 1 tablet of Truvada approximately 24 hours later
- Tablets need to be taken every 24 hours with a window period of 2 hours before or after the scheduled time.

All participants, irrespective of their regimen, can opt to switch regimen and to discontinue or to (re)start using PrEP at every FU visit at the HIV/STI clinic at ITM. Before being given a new supply, the participant must be confirmed to be HIV negative using HIV point-of-care tests.

### Study Visits

All participants are followed up for 18 months and will undergo a total of 9 prescheduled visits corresponding to screening, enrollment, FU month 1, and 6 3-monthly FU visits. During every prescheduled visit, participants see a social scientist (or social science assistant), a study nurse, and a physician.

Table 1 summarizes the study procedures. At the screening visit after written informed consent is obtained, study staff collects data on basic sociodemographics and current sexual behavior with special attention to high risk criteria (see Textbox 2); performs a physical examination with special attention to symptoms of an acute HIV infection; collects blood, urine, anal, and pharyngeal samples for kidney, liver, HIV, and STI testing; and performs preventive counseling.

The participant is invited to come back to the clinic within 2 weeks to confirm eligibility, including a reassessment of symptoms of acute HIV infection, relevant medical history, and current medication and recreational drug use. When eligible, the participant receives 1 box of 30 Truvada tablets, and the different PrEP regimens are discussed. Detailed information on PrEP use, adherence counseling, and preventive sexual health counseling is provided. Study staff explains the use of an online diary to collect data on sexual activity and adherence throughout study participation. The participant is also instructed to complete a self-administered questionnaire on a tablet computer.

At every follow-up visit, adverse events and concomitant medication are documented and a physical examination and HIV testing are performed. In addition, participants are screened for STIs every 3 months. Oral fluid samples are taken every 3 months using the Intercept i2 collection device (OraSure Technologies Inc) for future HIV testing. PrEP-related toxicity is monitored, adherence and prevention counseling is provided, and PrEP use is discussed. The participant takes the leftover Truvada back to the clinic and gets a refill to cover the needs until the next visit, with a maximum of 90 tablets. Intra- and extracellular drug level assessment of tenofovir or tenofovir diphosphate is performed at month 1 and month 3. Afterward, drug level monitoring will only take place for a proportion of daily users and some interesting event-driven users at the end of the study.

At FU month 15, specific counseling will address the impact of discontinuing PrEP use and will support participants in developing personal risk reduction solutions, since PrEP may not be available and reimbursed after individual completion of the study.

Different methods are being used to measure adherence: online diary, pill counts, questionnaire, and drug level assessment. Results of these measurements will be triangulated to assess adherence. When a participant is diagnosed with an STI, treatment will be given according to national guidelines.

**Table 1:** Schedule of assessments.

Procedures	Screening (1-2 weeks prior)	Enrollment (day 0)	FU^a^ month 1	FU month 3, 6, 9, 12, 15	FU month 18
Informed consent	X				
Relevant medical history		X			
Current/concomitant medication		X	X	X	X
Adverse events			X	X	X
Diary collection			X	X	X
HIV rapid test	X		X	X	X
HIV antigen test	X		X	X	X
Syphilis	X			X	X
HSV-2^b^	X			X^c^	X^d^
Hepatitis B	X				X
Hepatitis C	X			X^c^	X^d^
Creatinine	X			X	X
Phosphate	X				X
ALT/AST^e^	X				X
Proteinuria	X			X	X
CT/NG/MG/TV^f^ (urine)	X			X	X
Anorectal and pharyngeal swab for CT/NG/MG/TV	X			X	X
Oral fluid collection				X	X
Drug level testing (blood/hair)			X	X	X
Provide Truvada		X	X	X	
IDI^g^ (subsample of men)			X	X^h^	X
Questionnaire		X	X	X	X
Preventive sexual health counseling	X	X	X	X	X
Adherence counseling		X	X	X	X

^a^FU: follow-up.

^b^HSV-2: herpes simplex-2 virus.

^c^Will only be done using a look-back procedure when the final visit is positive to determine the time of infection more accurately, if funding permits.

^d^Only when screening visit result was negative.

^e^ALT/AST: alanine transaminase/aspartate transaminase.

^f^CT/NG/MG/TV: *Chlamydia trachomatis* / *Neisseria gonorrhoeae* / *Mycoplasma genitalium* / *Trichomonas vaginalis*.

^g^IDI: in-depth interview.

^h^Only month 9.

### Online Diary

The participant is asked to complete an online diary with information regarding pill intake, number of rectal and oral sex acts, and an individual risk assessment of acquiring HIV for each day participated in the study. A Web platform is created where participants can log in with a personal account using their smartphone, laptop, or other devices. They are instructed to complete the online diary every day or at least twice a week to limit recall bias. At every visit, and more clearly emphasized at the month 1 visit, the social scientist or designee examines all diaries for completion to allow for optimal data collection and verifies whether participants encountered problems or difficulties or unclear procedures when completing the online diary. Participants also receive an email when they have not completed their diary for more than 7 days. Event-driven users are able to complete the diary only during sexual active periods. In addition, a paper version is available if preferred. Finally, an audit trail is available.

### Questionnaire

At the enrollment visit, a detailed electronic self-administered and standardized questionnaire collects data on sociodemographic characteristics, well-being, sexual lifestyle, sexual behaviors, and determinants related to HIV risk, as well as motivations for (not) choosing either PrEP dosing regimen. It was based on similar questionnaires used in other PrEP studies or HIV prevention and sexual behavior research among MSM. The questionnaire was pilot-tested before study initiation among 7 MSM, and their suggestions were incorporated into the final version. It was translated and back-translated in 3 different languages: Dutch, French, and English. At the FU visits, a shortened version is used assessing adherence, recent sexual behavior, and reasons for switching PrEP dosing regimen if applicable. At FU month 9, a more comprehensive questionnaire is used reassessing several measures of the enrollment questionnaire (eg, well-being). At FU month 18, a questionnaire is used similar to FU month 9 that includes questions assessing participant experiences of and attitudes toward using and receiving PrEP, for which the content will be informed by the in-depth interviews (IDIs) and their experiences of study participation such as the collection of oral fluid for HIV testing.

### In-Depth Interviews

To explore participant prevention needs, their preferences for and attitudes toward PrEP use (ie, regimen choice and decision making), user experiences, and perceived influences on their sex lives, 35 to 40 IDIs are conducted throughout the project. All interviews are conducted by a social scientist with expertise in qualitative research after obtaining informed consent. The data collection and analysis is guided by an inductive approach based on grounded theory [19,20]. The topic guide is developed within the study team and is amended where necessary to improve data collection and account for an iterative qualitative data collection approach without losing consistency [21]. Dutch- or English-speaking participants are purposely selected based on information-rich events (eg, switching PrEP regimen) and availability. Triangulating the results of the IDIs with other quantitative data from the trial will allow for improving validity of the overall study results.

### Laboratory Procedures

Table 2 provides an overview of all laboratory tests that are performed in the study. All testing is performed at ITM except for drug level testing which is performed at the University Hospital of Gent, Belgium. Dry blood spots (DBS) are taken at every visit where blood is taken and stored together with oral fluid and hair samples (when consented) for future HIV testing or drug level testing.

**Table 2:** Be-PrEP-ared laboratory procedures.

To be tested	Kind of test
HIV	See Figure 2
Syphilis	RPR^a^ (Macro-Vue, BD) and TPA (Vitros 5600) /TPPA^b^ (Fujirebio)
Hepatitis B	HBsAg/HBsAb^c^, HBcIg/HBcIgM^d^ (Vitros 5600)
Hepatitis C	Antibody Hepatitis C (Vitros 5600)
Biochemistry: AST/ALT^e^, creatinine, and phosphorus	Creatinine clearance calculated using CKD-Epi^f^ formula (Vitros 5600)
Proteinuria	Urine dipstick (Siemens Hema-Combistix)
*Chlamydia trachomatis (CT)* / *Neisseria gonorrhoeae (NG)*	Abbott Real Time *CT* / *NG* with confirmation by in-house PCR^g^
*Mycoplasma genitalium (MG)* / *Trichomonas vaginalis (TV)*	In-house PCR for *MG* and Diagenode (S-DiaMGTV) for *TV*
Herpes simplex virus-2 (HSV-2)	Kalon HSV Type 2 IgG
Plasma and upper layer packed cell drug level testing	Thermo Scientific Q-Exactive hybrid quadrupole–Orbitrap mass spectrometer (LC-MS/MS^h^ system)
HIV-1 resistance	RNA sequencing
HIV-1 viral load	Cobas 4800 (Roche)

^a^RPR: rapid plasma reagin.

^b^TPA/TPPA: *Treponema pallidum* assay/ *Treponema pallidum* particle agglutination assay.

^c^HBsAg/HBsAb: hepatitis B surface antigen/hepatitis B surface antibody.

^d^HBcIg/HBcIgM: total hepatitis B core antibody/hepatitis B core IgM antibody.

^e^AST/ALT: aspartate transaminase/alanine transaminase.

^f^CKD-Epi: Chronic Kidney Disease Epidemiology Collaboration.

^g^PCR: polymerase chain reaction.

^h^LC-MS/MS: liquid chromatography coupled with tandem mass spectrometry.

**Figure 2:**
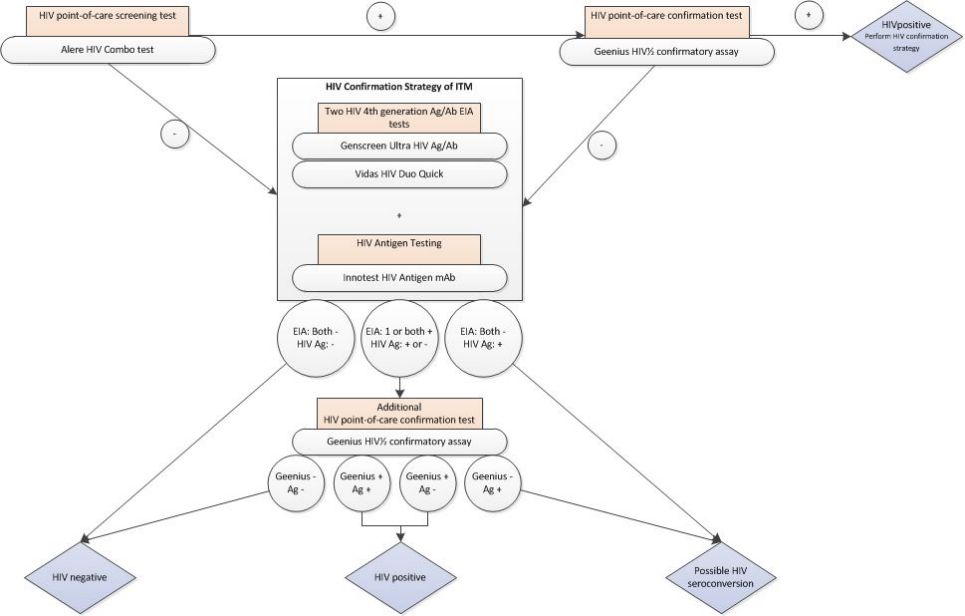
HIV algorithm in the study.

### HIV Seroconverter Procedures

If a participant becomes HIV positive during the study, he is discontinued immediately from the study but is followed up for safety. In this case, study staff collects all unused pills, conducts the final visit procedures including resistance and viral load testing, discusses the pros and cons of early ARV therapy, and refers the participant to an AIDS reference center of choice for linkage to HIV care.

### Safety

Safety and tolerability of Truvada is evaluated by recording adverse events (AEs) and grading laboratory and vital signs evaluations in the electronic case report form (eCRF) starting from enrollment until the final visit. Severity, causality, and outcome are also assessed by the study physician. Any event that occurred before the enrollment visit is documented as medical history. All AEs are followed up until resolution to the extent possible. An HIV infection is not considered a serious adverse event (SAE). Due to the possible renal adverse effects of Truvada, elevations in serum creatinine are monitored closely. Truvada will be interrupted when creatinine clearance is below 60 mL/minute/1.73 m² and will be permanently discontinued when the clearance stays below 50 mL/minute/1.73 m² after repeat testing.

All SAEs whether or not deemed drug-related or expected are reported within 24 hours (1 working day) to the sponsor (ITM). Line listings of all reported SAEs are sent to the concerned ethics committee (EC) and the Belgian Competent Authority (CA) on a yearly basis. In addition, all fatal or life-threatening suspected unexpected serious adverse reactions (SUSARs) need to be reported to the Belgian CA and to the concerned EC within 7 days. Nonfatal and non–life-threatening SUSARs must be reported within 15 days. Gilead Sciences is notified immediately in case of SUSARs and will receive SAE listings every 2 weeks.

No formal data safety monitoring board (DSMB) has been set up due the fact that this drug is widely used and approved for treatment and prevention of HIV infection by the FDA and EMA. However, an independent data safety monitor has been appointed to review all SAE reports. In case of major safety concerns, this monitor may advise the sponsor to halt recruitment of the trial and/or organize a formal DSMB with a complete overview of the available safety data.

### Data Collection

#### Databases

Due to the project’s mixed methods approach, 4 different types of databases have been set up:

- A clinical trial database was programmed and validated prior to project start: an eCRF developed in the Good Clinical Practice (GCP)-compliant clinical trial software MACRO (InferMed, United Kingdom) with CFR 21 Part 11 in-built consistency checks is used.
- An online survey database is used for the questionnaires (using Survey to Go mobile survey software from Dooblo for the development of the online questionnaires).
- The online diary data is stored on a secured and password-protected Web platform that was created for the purpose of this project.
- The interview data from the IDIs is stored using a computer-assisted software program for data storage and analysis (NVivo 10.0, QSR International).

#### Confidentiality and Security of Trial Participant Data

Private information on trial participants is handled confidentially. Only the participant identification number, initials, and date of birth are captured in the eCRF and all other study documentation. Name and contact data for each participant is kept separately, and access to them is limited to the authorized study staff. The same confidentiality rules apply for all study documents and electronic files. The computers and eCRFs are only accessible by the study staff with personal username and password. The online diary and survey data are stored on secured servers only accessible to the researchers.

### Data Analysis

#### End Points

The uptake, acceptability, and feasibility of using PrEP will be examined by the end points documented in Textbox 3.

**End points of the Be-PrEP-ared study.**

Preventive needs:

- Recruitment rate (number of screened participants/number of registered on study-specific website)
- (Un)safe sex behavior during the last 6 months
- Percentage of reported intention to use pre-exposure prophylaxis (PrEP) in the future at final visit
- Retention rates in the different regimens
- Attitudes towards PrEP use: satisfaction and motivation for future use

Adherence:

- (In)consistent pill take, percentage of days with no pill taken/days on which a pill should have been taken
- Tenofovir drug levels in blood and/or hair samples
- Perceived skills to adhere, including self-efficacy

Impact of PrEP use on other preventive strategies:

- Number of sex partners
- Self-reported condom use
- Sex under influence (alcohol, drugs)

Impact of PrEP use on sexually transmitted infection (STI) trends (descriptive analyses only):

- STI incidence and trends: *Chlamydia trachomatis* / *Neisseria gonorrhoeae*, *Mycoplasma genitalium* / *Trichomonas vaginalis*, herpes simplex virus-2, syphilis, and hepatitis C

Safety of the different regimens of PrEP use:

- Rate of adverse events related to PrEP

Real-life effectiveness of PrEP use:

- Incidence of HIV infection by regimen
- Genotypic viral resistance

Feasibility of oral fluid self-sampling testing:

- Feasibility

A mixed method analysis of quantitative and qualitative end points will be conducted by triangulating the results from both data collection types.

#### Clinical Data Analysis

The study design is observational, and all analyses will be descriptive. As participants may change regimens, a single participant may be included in different regimens over time and contribute to person-months of daily, event-driven, or no use in the analyses.

Adherence may be dichotomized as adherent/nonadherent and is defined in general as the proportion of pills the participant needed to take that were actually taken. The number of pills actually taken is calculated based on self-reporting through the diary. Different indicators for adherence will be used for each of the regimens and per 3 months:

- Adherence to regimen: number of pills taken/number of pills which should have been taken according to the PrEP dosing regimen.
- Estimated proportion of covered sex acts: number of sex acts covered with PrEP/number of sex acts. This indicator will be calculated separately for high-risk sexual activities and low-risk sexual activities. High-risk sexual activities are defined as anal intercourse without a condom with a partner of unknown HIV status or known to be HIV positive with detectable virus load.

Exploratory analysis of predictors of retention on PrEP or (non)adherence may be performed using regression models (linear, logistic, or Cox-regression). Different groups may be considered for analysis depending on the change of regimen: group remaining on daily use, group remaining on event-driven, group changing from daily to event-driven, and group changing from event-driven to daily use.

All nonserious and serious AEs will be grouped according to a prespecified side-effect coding system and tabulated. The number of subjects experiencing any AE, any SAE, and any drug-related SAE will be summarized by PrEP regimen.

#### Questionnaire Data Analysis

Analysis of the questionnaire data will be mainly descriptive, using uni- and bivariate statistical analyses. Depending on the results, multivariable statistical models and longitudinal analyses may be used. All computations will be done using SPSS version 23.0 (IBM Corp).

#### Interview Data Analysis

Interview data will be analyzed inductively based on grounded theory principles using multiple, independent coders. They will establish a data-driven codebook. This approach ensures triangulation from different perspectives [19,20] and thus improves data validity.

### Ethics and Quality Assurance

The protocol and all study documents were reviewed and approved by the Institutional Review Board (IRB) of the ITM, the EC of the University Hospital of Antwerp, and the CA of Belgium. No study activities were performed before approval from all these bodies. Amendments to the protocol must be approved by the sponsor and by the concerned IRB, EC, and CA. Yearly updates are sent to all of these bodies. The study is carried out according to the principles stated in the Declaration of Helsinki as amended in 2013 and any further updates, all applicable national and international regulations, and according to the most recent International Conference on Harmonization (ICH) and WHO GCP guidelines. All laboratory activities are conducted in accordance with Good Clinical Laboratory Practices (GCLP) and EN-ISO (International Organisation for Standardization) 15189.

The study is monitored in accordance with regulations applicable to clinical trials, including ICH-GCP and GCLP requirements, and sponsor-specific monitoring and source data verification standard operating procedures. A trial management group is in charge of the day-to-day management of the clinical study.

### Informed Consent

Before any study procedures took place, participants were asked to provide written informed consent.

### Community Advisory Board

A Community Advisory Board (CAB) was set up with representatives from local and regional MSM community and health organizations and Belgian MSM prevention experts to ensure that the demonstration project meets the target group’s needs. The researchers consult with this CAB twice yearly or more often if needed. Its main purposes are to assist in recruiting participants; to ensure proper feedback of the project results to the community; and to assist in safeguarding the community’s ethical, social, and cultural norms.

## Results

The clinical trial part of the study has been registered in the EudraCT database (EudraCT 2015-000054-37). A total of 200 participants were enrolled on December 12, 2016. The last participant’s last visit will take place around Q2 2018.

## Discussion

Given EMA’s recent approval of PrEP and recent national developments, we assume that PrEP will soon be available and may even be (partially) reimbursable in Belgium. Our study results will be useful for PrEP implementation as part of overall HIV combination prevention (eg, screening guidelines for PrEP eligibility). Given the mixed method approach and longitudinal data collection, this study will provide insights into factors influencing PrEP use and choice of regimen—as participants are able to switch dosing regimen or to discontinue—and how it relates to user perspectives. Actual PrEP use may shift and be influenced by several factors such as users’ self-perceived risk for HIV, actual user experience with PrEP, PrEP adherence, and perceived impact on sexuality. These findings could have important implications for HIV prevention policies and health care expenditure.

To our knowledge, the Be-PrEP-ared study is, together with the AMPrEP project of Amsterdam, the first study investigating the uptake of different PrEP dosing regimens at the choice of the participant [22]. Moreover, the study is novel in longitudinally exploring the preferences for and experiences of using the different regimens or discontinuing and restarting PrEP. Allowing participants to adapt PrEP use and different regimens to better suit periods of perceived high risk of HIV infection is novel and could lead to important insights into the need for PrEP within this high-risk population. Furthermore, such insights are crucial for developing appropriate adherence counseling guidelines and an optimal integration of PrEP within the already existing tools for HIV prevention.

One major strength of this project is its strong mixed methods approach, allowing for methodological triangulating of the various data sources and for exploring MSM’s prevention needs in depth. This will lead to an improved understanding of the uptake and acceptability of PrEP use within the current context and greatly improves the validity of the results [23].

Moreover, the mixed methods approach will lead to important in-depth insights about and knowledge of adherence to PrEP, one of the key outcomes of the project. In our study we combine different methods to assess adherence to PrEP. We test drug levels of tenofovir and tenofovir diphosphate immediately in plasma and upper layer packed cells at FU month 1 and FU month 3. With intracellular tenofovir diphosphate, an assessment of the adherence over the past 2 to 4 weeks can be made and “white-coat adherence” (ie, only taking PrEP just before the study visit) can be detected [24]. In addition, DBS and hair are also stored for future emtricitabine/tenofovir disoproxil fumarate therapeutic drug monitoring testing to reflect longer term windows of exposure when funding is available. Furthermore, the online diary will lead to improved insights into the number and timing of pills taken throughout the study, whereas the questionnaire and online diary will explore attitudes towards adherence to the medication.

Another strength is the participation of and close communication with health care providers and members of the HIV prevention and MSM community in Belgium through the CAB. The use of the CAB not only helps to raise awareness about the study and to more efficiently disseminate the results of the study, it is also crucial in developing study procedures and implementation guidelines that are sensitive to those who will be using PrEP [25,26].

The number of MSM who registered for participation in the first 3 weeks of launching the registration study website (ie, 196 in total) shows that there is an interest for PrEP within this population. However, interpreting the registration rate as a measure for PrEP acceptability would remain difficult: we did not overly promote study registration after the initial 3 weeks as it was clear that the desired number of participants would be reached; not all possible candidates may thus have been aware of the study and some may have anticipated not being able to participate in the study due to the promoted consultation hours (ie, during office hours) or may have been unwilling due to setting-related reasons (eg, distance to the clinic).

The slow recruitment rate was mainly due to limited staff resources (ie, limited availability of study physicians) resulting in approximately 4 screenings per week. This could be an important limitation, as those enrolled first could differ from those enrolled last (ie, as PrEP use is at least 1 year apart) and thus would not be comparable. Media coverage or PrEP availability, changing sexual norms within the community, and related factors may have had a different influence on participants. However, the study may reflect actual willingness to use PrEP and allows for exploring how an evolving HIV prevention landscape including access to PrEP affects individual PrEP uptake and adherence. Moreover, it cannot be excluded that individual differences between the start and end of the study will be the result of similar influences rather than PrEP use on its own.

Conducting a demonstration project such as Be-PrEP-ared may in itself have an impact on HIV prevention that goes beyond providing ARV medication to participants. It has reinforced collaborations with community organizations and health care providers and can help in increasing PrEP awareness and influence policy on HIV prevention. In the wake of Be-PrEP-ared, various substudies have already started in Belgium such as a survey among health care providers to assess their PrEP knowledge, attitudes, and willingness to prescribe. New studies are being set up in this evolving field, which will be important to allow for crossnational research. To develop a good understanding of how to optimally implement and provide PrEP integrated into the existing health care structures, new research will be of paramount importance.

In conclusion, results from this study will contribute to a better understanding of PrEP users’ experiences including their choices for specific PrEP regimens. The findings will help to inform appropriate delivery strategies for the roll-out of PrEP and for policy makers to consider financial reimbursement of PrEP in Belgium.

## Abbreviations

AE: adverse event
ALT/AST: alanine transaminase/aspartate transaminase
ARV: antiretroviral
CA: Competent Authority
CAB: Community Advisory Board
CT: Chlamydia trachomatis
DBS: dry blood spot
DSMB: data and safety monitoring board
EC: ethics committee
eCRF: electronic case report form
EMA: European Medicines Agency
FDA: Food and Drug Administration
FU: follow-up
GCLP: Good Clinical Laboratory Practices
GCP: Good Clinical Practices
HBV: hepatitis B virus
HCV: hepatitis C virus
HSV-2: herpes simplex-2 virus
ICH: International Conference on Harmonization
IDI: in-depth interview
IRB: Institutional Review Board
ISO: International Organisation for Standardization
ITM: Institute of Tropical Medicine
MSM: men who have sex with men
MG: Mycoplasma genitalium
NG: Neisseria gonorrhoeae
PrEP: pre-exposure prophylaxis
SAE: serious adverse event
STI: sexually transmitted infection
SUSAR: suspected unexpected serious adverse reaction
TV: Trichomonas vaginalis
WHO: World Health Organization
